# Synthesis, characterization and crystal structure of a 2-(diethylaminomethyl)indole ligated dimethyl­aluminium complex

**DOI:** 10.1107/S2056989015017053

**Published:** 2015-09-26

**Authors:** Logan E. Shephard, Nicholas B. Kingsley

**Affiliations:** a Department of Chemistry and Biochemistry, 556 MSB, 303 E. Kearsley, Flint, MI 48502, USA

**Keywords:** crystal structure, aluminium, indol­yl, C—H⋯π inter­actions

## Abstract

The title compound, [Al(CH_3_)_2_(C_13_H_17_N_2_)] (systematic name; {2-[(di­ethyl­amino)­meth­yl]indol-1-yl-κ^2^ *N*,*N*′}di­methyl­aluminium), was prepared by methane elimination from the reaction of 2-(di­ethyl­amino­meth­yl)indole and tri­methyl­aluminium. The complex crystallizes readily from a concentrated toluene solution in high yield. The asymmetric unit contains two crystallographically independent mol­ecules. Each mol­ecule has a four-coordinate aluminium atom that has pseudo-tetra­hedral geometry. C—H⋯π inter­actions link the independent mol­ecules into chains extending along the *b*-axis direction.

## Abstract 2

The title aluminium complex was prepared by methane elimination from the reaction of 2-(di­ethyl­amino­meth­yl)indole and tri­methyl­aluminium. Each of the two crystallographically independent mol­ecules has a four-coordinate aluminium center that has pseudo-tetra­hedral geometry.

## Chemical context   

Organoaluminium chemistry has a long history of active research that has led to numerous applications in industry (Mason, 2005 ▸). Organoaluminium compounds have garnered much attention in recent years for their use in the formation of polyactides, (Liu *et al.*, 2010 ▸; Chisholm *et al.*, 2003 ▸, 2005 ▸; Zhang *et al.*, 2014 ▸; Chen *et al.*, 2012 ▸; Schwarz *et al.*, 2010 ▸) and hydro­amination (Koller & Bergman, 2010*a* ▸,*b* ▸; Khandelwal & Wehmschulte, 2012 ▸). While many varieties of ancillary ligands on aluminium have been employed in such reactions, a majority of these systems have nitro­gen-donor arms as a component. Our group is inter­ested in particular in the use of 2-(di­alkyl­amino­meth­yl)indoles (Nagarathnam, 1992 ▸) as ligands for organoaluminium complexes. Herein we report the synthesis, characterization and crystal structure of the first 2-(di­alkyl­amino­meth­yl)indol­yl–aluminium complex, [Al(CH_3_)_2_(C_13_H_17_N_2_)].
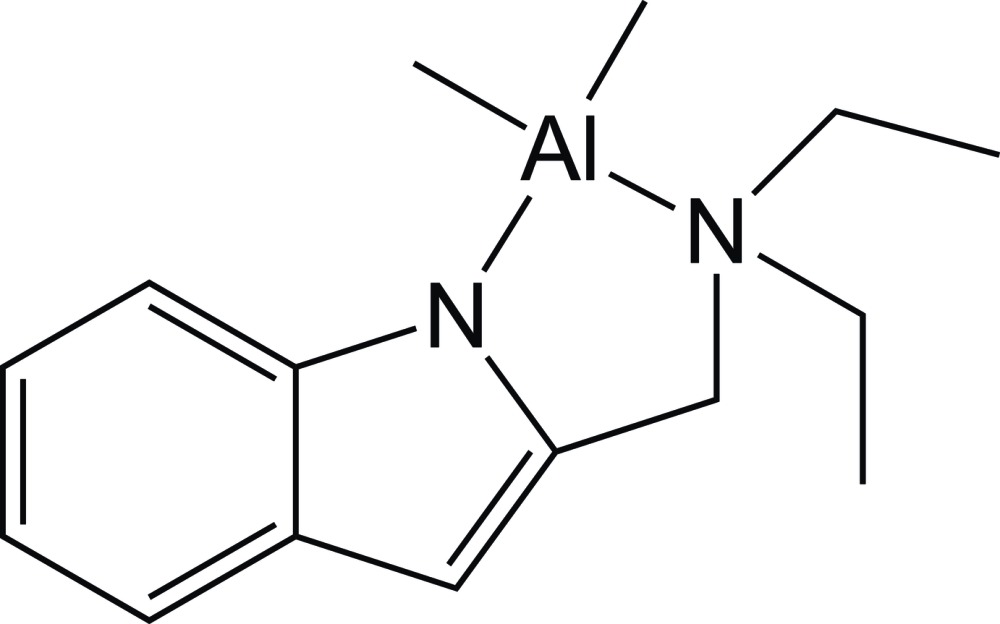


## Structural commentary   

**Figure 1:**
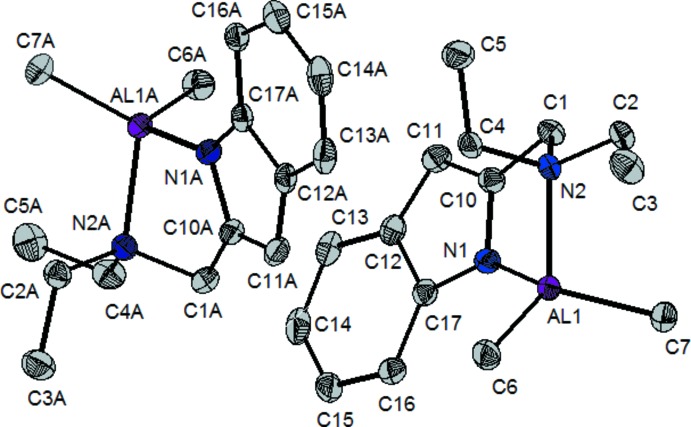
A view of the asymmetric unit of the title compound, showing the atom labeling. Displacement ellipsoids are drawn at the 50% probability level. H atoms have been omitted for clarity.

The asymmetric unit of the title complex contains two independent mol­ecules (Fig. 1 ▸). They are structurally different with regard to the chelate rings that are formed around the aluminium atoms by the indolyl moiety. The most obvious difference between the two crystallographically independent mol­ecules is the displacement of the Al atom from the plane of the chelate ring. Al1 deviates by 0.6831 (5) Å from the plane defined by atoms N1/C10/C1/N2 while Al1*A* deviates by 0.6150 (5) Å from the plane N1*A*/C10*A*/C1*A*/N2*A*. Each mol­ecule contains a four-coordinate, pseudo-tetra­hedral, aluminium atom. There are two distinct bond lengths for the Al—N bonds in the mol­ecule. The Al—N_indol­yl_ bond lengths are 1.8879 (14) Å for Al1—N1 and 1.8779 (15) Å for Al1*A*—N1*A*. These lengths are in the range expected for anionically bound indolyl or pyrrolyl moieties (Huang *et al.*, 2001 ▸). As expected, these lengths are significantly shorter than those found for the dative Al—N_imine_ bonds, 2.0355 (15) Å for Al1—N2 and 2.0397 (16) Å for Al1*A*—N2*A* [see Huang *et al.* (2001 ▸) for typical values].

## Supra­molecular features   

**Figure 2:**
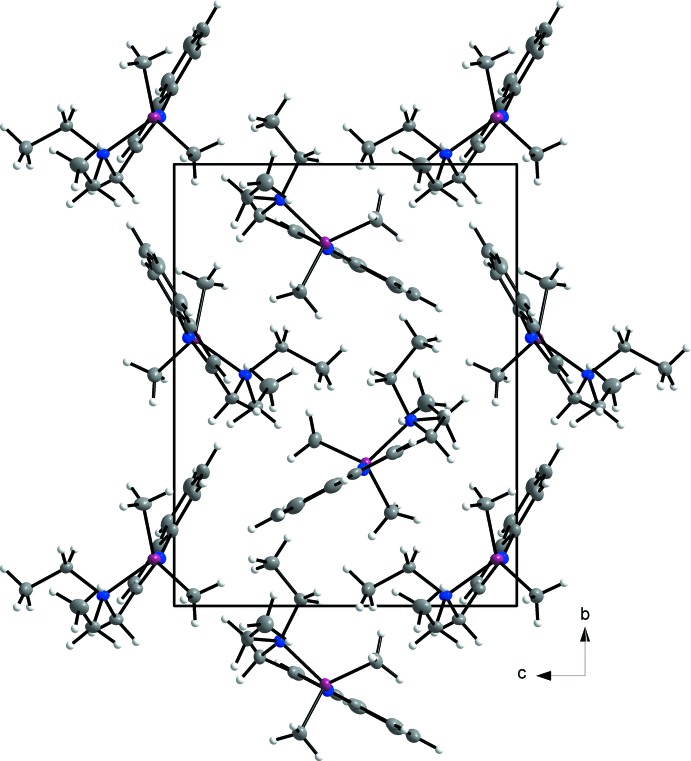
Crystal packing diagram of the title compound viewed along the *a* axis.

**Figure 3:**
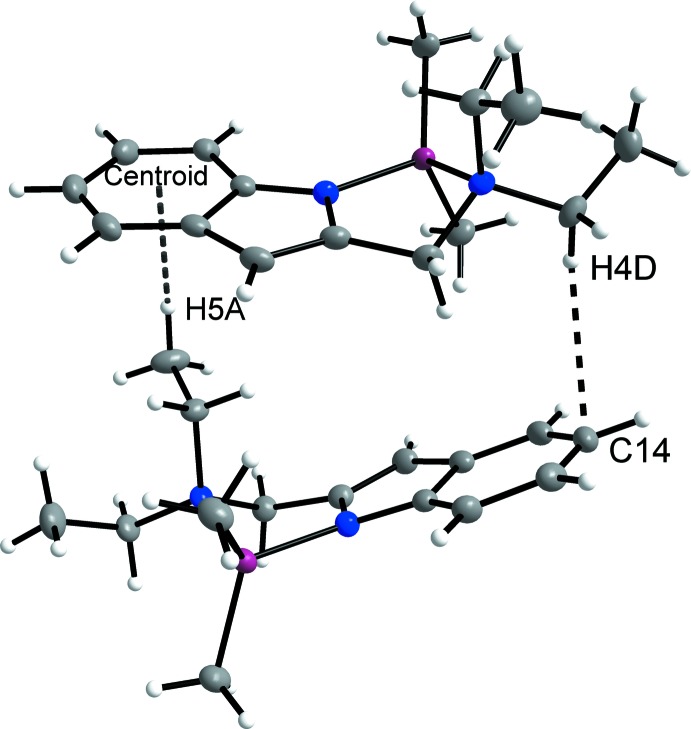
C—H⋯π inter­actions between mol­ecules in the asymmetric unit.

The crystal packing is illustrated in Fig. 2 ▸. In the crystal, mol­ecules associate *via* three different types of C—H⋯π inter­actions, as shown in Figs. 3 ▸ and 4 ▸. There is one inter­action between the methyl proton H5*A* and the centroid of the (C12*A*–C17*A*) aromatic ring of 2.57 Å (Table 1 ▸) and another between the methyl­ene proton H4*D* and the aromatic C14 of 2.88 Å. The third inter­action is between H2*B* and the centroid of C12*A* ^i^–C17*A* ^i^ [Table 1 ▸; symmetry code: (i) 1 − *x*, −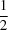 + *y*, 1 − *z*]. This inter­action links the two independent mol­ecules in the asymmetric unit into chains that extend along the *b-*axis direction.

## Database survey   

A search of the Cambridge Structural Database (CSD, Version 5.36; Groom & Allen, 2014 ▸) for indolyl gave 500 hits. A search for indolide generated 18 hits. Neither of these sets of hits included structures involving indolyl moieties bound to aluminium. A substructure search for N-bound indolyl-coordinating aluminium complexes resulted in only five hits (Kingsley *et al.*, 2010 ▸), all of which contained bridging μ^2^:η^1^:η^1^ coordination modes. The title compound is the first struct­urally characterized complex with a monomeric μ^1^:η^1^-coordinating indole moiety to aluminium.

## Synthesis and crystallization   

To a 100 mL side-arm flask was added 2-(di­ethyl­amino­meth­yl)indole (0.402 g, 2.0 mmol) and 25 mL of toluene. A toluene solution of tri­methyl­aluminium (1.0 mL, 2.0 *M*, 2.0 mmol) was added *via* syringe. The reaction solution turned bright yellow, which darkened as the solution was stirred for 12 h. The solvent was then removed *in vacuo* resulting in a yellow solid, which was dissolved in a mixture of 10 mL of hot toluene, followed by cooling to 243 K for 48 h. The resulting yellow crystalline material was isolated by filtration. Yield: 0.462 g, 1.78 mmol, 90%. ^1^H NMR (CDCl_3_, 600 MHz): δ 7.55 (*d*, ^3^ *J*_HH_ = 7.8 Hz, 1H, H16), 7.36 (*d*, ^3^ *J*_HH_ = 7.8 Hz, 1H, H13), 7.07 (*t*, ^3^ *J*_HH_ = 7.8 Hz, 1H, H15), 7.00 (*t*, ^3^ *J*_HH_ = 7.8 Hz, 1H, H14), 6.31 (*s*, 1H, H11), 4.00 (*s*, 2H, indole CH_2_), 2.88 (*q*, ^3^ *J*_HH_ = 7.2 Hz, 4H, amino *CH_2_*CH_3_), 1.13 (*t*, ^3^ *J*_HH_ = 7.2 Hz, 6H, amino CH_2_ *CH_3_*), −0.59 (*s*, 6H, AlCH_3_). ^13^C{^1^H} NMR (CDCl_3_, 150.8 MHz): δ 141.7 (C17), 139.4 (C10), 131.8 (C12), 120.2 (C15), 119.6 (C16), 118.5 (C15), 113.7 (C14), 98.1 (C11), 53.2 (indole *C*H_2_), 44.7 (amino *CH_2_*CH_3_), 8.3 (amino CH_2_ *CH_3_*), −11.10 (*br*, Al*C*H_3_) (Kingsley *et al.*, 2010 ▸). Analysis calculated for C_15_H_23_N_2_Al: C, 69.74; H, 8.97; N, 10.84. Found: C, 69.67; H, 8.70; N, 10.63.
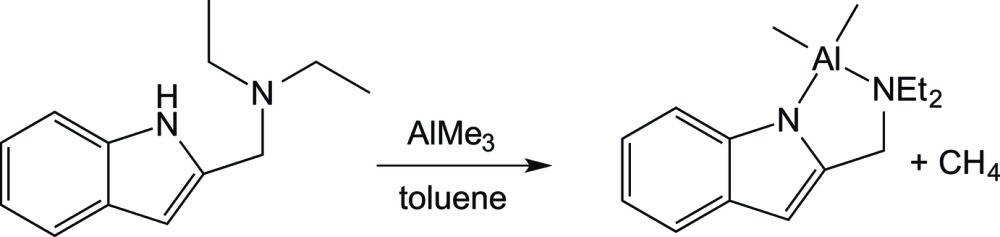


X-ray quality crystals were grown from a concentrated solution in hot toluene followed by slow cooling to room temperature followed by storage at 243 K for 72 h.

## Refinement   

Crystal data, data collection and structure refinement details are summarized in Table 2 ▸. All H atoms were positioned geometrically and refined using a riding model with C—H = 0.05–0.99 Å and *U*_iso_(H) = 1.2 or 1.5*U*_eq_(C).

## Supplementary Material

Crystal structure: contains datablock(s) I. DOI: 10.1107/S2056989015017053/zl2630sup1.cif

Structure factors: contains datablock(s) I. DOI: 10.1107/S2056989015017053/zl2630Isup2.hkl

CCDC reference: 1423793

Additional supporting information:  crystallographic information; 3D view; checkCIF report

## Figures and Tables

**Figure 4:**
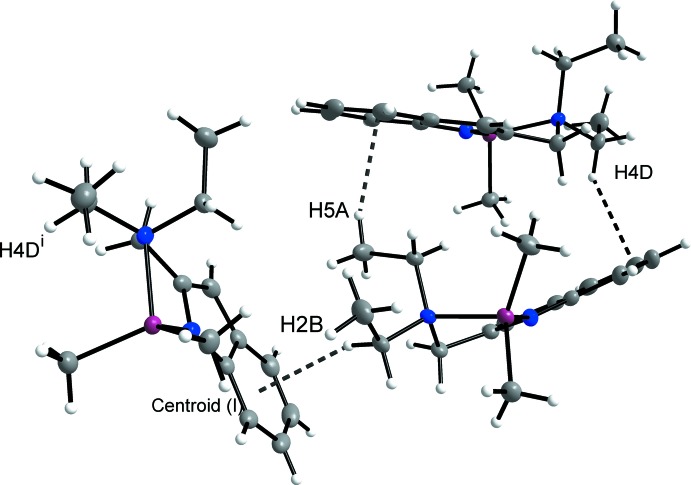
All C—H⋯π inter­actions between mol­ecules of the title compound. [Symmetry code: (i) 1 − *x*, −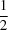 + *y*, 1 − *z*.]

**Table 1:** CH interactions (, ) *Cg*1 is the centroid of the C12*A*C17*A* ring.

*D*H*A*	*D*H	H*A*	*D* *A*	*D*H*A*
C5H5*A* *Cg*1	0.98	2.57	3.470(2)	153
C2H2*B* *Cg*1^i^	0.99	2.55	3.434(2)	149

Symmetry code: (i) 
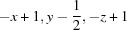
.

**Table 2:** Experimental details

Crystal data	
Chemical formula	[Al(CH_3_)_2_(C_13_H_17_N_2_)]
*M*_r_	258.33
Crystal system, space group	Monoclinic, *P*2_1_
Temperature (K)	150
*a*, *b*, *c* ()	9.7467(5), 14.1245(7), 10.9866(5)
()	94.206(1)
*V* (^3^)	1508.42(13)
*Z*	4
Radiation type	Mo *K*
(mm^1^)	0.12
Crystal size (mm)	0.20 0.20 0.15

Data collection	
Diffractometer	Bruker APEXII CCD
Absorption correction	Multi-scan (*SADABS*; Bruker, 2003 ▸)
*T*_min_, *T*_max_	0.697, 0.745
No. of measured, independent and observed [*I* > 2(*I*)] reflections	13157, 5440, 5366
*R*_int_	0.025
(sin /)_max_ (^1^)	0.624

Refinement	
*R*[*F* ^2^ > 2(*F* ^2^)], *wR*(*F* ^2^), *S*	0.024, 0.068, 1.05
No. of reflections	5440
No. of parameters	333
No. of restraints	1
H-atom treatment	H-atom parameters constrained
_max_, _min_ (e ^3^)	0.21, 0.19
Absolute structure	Flack *x* determined using 2203 quotients [(*I* ^+^)(*I* )]/[(*I* ^+^)+(*I* )] (Parsons *et al.*, 2013 ▸)
Absolute structure parameter	0.05(3)

Computer programs: *APEX2* (Bruker, 2005 ▸), *SAINT* (Bruker, 2003 ▸), *SHELXS97* (Sheldrick, 2008 ▸), *SHELXL2014* (Sheldrick, 2015 ▸), *DIAMOND* (Brandenburg, 2010 ▸) and *publCIF* (Westrip, 2010 ▸)..

## supplementary crystallographic information

### Crystal data

**Table:**

[Al(CH_3_)_2_(C_13_H_17_N_2_)]	*F*(000) = 560
*M**_r_* = 258.33	*D*_x_ = 1.138 Mg m^−^^3^
Monoclinic, *P*2_1_	Mo *K*α radiation, λ = 0.71073 Å
*a* = 9.7467 (5) Å	Cell parameters from 5904 reflections
*b* = 14.1245 (7) Å	θ = 2.4–26.4°
*c* = 10.9866 (5) Å	µ = 0.12 mm^−^^1^
β = 94.206 (1)°	*T* = 150 K
*V* = 1508.42 (13) Å^3^	Irregular, yellow
*Z* = 4	0.20 × 0.20 × 0.15 mm

### Data collection

**Table:**

Bruker APEXII CCD diffractometer	5366 reflections with *I* > 2σ(*I*)
Radiation source: sealed tube	*R*_int_ = 0.025
φ and ω scans	θ_max_ = 26.3°, θ_min_ = 1.9°
Absorption correction: multi-scan (*SADABS*; Bruker, 2003)	*h* = −12→10
*T*_min_ = 0.697, *T*_max_ = 0.745	*k* = −16→17
13157 measured reflections	*l* = −13→12
5440 independent reflections	

### Refinement

**Table:**

Refinement on *F*^2^	Hydrogen site location: inferred from neighbouring sites
Least-squares matrix: full	H-atom parameters constrained
*R*[*F*^2^ > 2σ(*F*^2^)] = 0.024	*w* = 1/[σ^2^(*F*_o_^2^) + (0.0388*P*)^2^ + 0.2513*P*] where *P* = (*F*_o_^2^ + 2*F*_c_^2^)/3
*wR*(*F*^2^) = 0.068	(Δ/σ)_max_ < 0.001
*S* = 1.05	Δρ_max_ = 0.21 e Å^−^^3^
5440 reflections	Δρ_min_ = −0.19 e Å^−^^3^
333 parameters	Absolute structure: Flack *x* determined using 2203 quotients [(*I*^+^)-(*I*^-^)]/[(*I*^+^)+(*I*^-^)] (Parsons *et al.*, 2013)
1 restraint	Absolute structure parameter: 0.05 (3)

### Special details

**Table:**

Geometry. All e.s.d.'s (except the e.s.d. in the dihedral angle between two l.s. planes) are estimated using the full covariance matrix. The cell e.s.d.'s are taken into account individually in the estimation of e.s.d.'s in distances, angles and torsion angles; correlations between e.s.d.'s in cell parameters are only used when they are defined by crystal symmetry. An approximate (isotropic) treatment of cell e.s.d.'s is used for estimating e.s.d.'s involving l.s. planes.

### Fractional atomic coordinates and isotropic or equivalent isotropic displacement parameters (Å^2^)

**Table:**

	*x*	*y*	*z*	*U*_iso_*/*U*_eq_	
Al1	0.49235 (5)	0.10530 (4)	0.05845 (4)	0.01712 (12)	
Al1A	0.00034 (5)	0.32156 (4)	0.44114 (4)	0.01652 (12)	
N1	0.29822 (14)	0.10862 (11)	0.04241 (13)	0.0187 (3)	
N1A	0.19261 (14)	0.30931 (11)	0.44801 (12)	0.0185 (3)	
N2	0.46863 (14)	0.02289 (10)	0.20745 (13)	0.0166 (3)	
N2A	0.02362 (15)	0.42016 (11)	0.30859 (12)	0.0185 (3)	
C1	0.33910 (17)	−0.03044 (13)	0.16893 (16)	0.0194 (3)	
H1A	0.3588	−0.0811	0.1105	0.023*	
H1B	0.3005	−0.0598	0.2408	0.023*	
C2	0.58090 (17)	−0.04771 (13)	0.23735 (16)	0.0210 (4)	
H2A	0.5922	−0.0876	0.1647	0.025*	
H2B	0.5532	−0.0896	0.3035	0.025*	
C3	0.7178 (2)	−0.00237 (15)	0.2769 (2)	0.0311 (4)	
H3A	0.7883	−0.0516	0.2893	0.047*	
H3B	0.7099	0.0322	0.3534	0.047*	
H3C	0.7439	0.0417	0.2136	0.047*	
C4	0.44570 (18)	0.08681 (13)	0.31419 (15)	0.0201 (3)	
H4A	0.3664	0.1285	0.2912	0.024*	
H4B	0.5276	0.1278	0.3291	0.024*	
C5	0.4190 (2)	0.03699 (15)	0.43275 (17)	0.0319 (4)	
H5A	0.4053	0.0843	0.4960	0.048*	
H5B	0.4981	−0.0030	0.4585	0.048*	
H5C	0.3365	−0.0025	0.4202	0.048*	
C6	0.57142 (19)	0.23092 (14)	0.09103 (18)	0.0263 (4)	
H6A	0.6534	0.2252	0.1478	0.039*	
H6B	0.5035	0.2713	0.1272	0.039*	
H6C	0.5969	0.2592	0.0144	0.039*	
C7	0.58197 (19)	0.02303 (15)	−0.05668 (16)	0.0254 (4)	
H7A	0.6789	0.0143	−0.0284	0.038*	
H7B	0.5760	0.0524	−0.1377	0.038*	
H7C	0.5357	−0.0386	−0.0613	0.038*	
C10	0.23890 (17)	0.03923 (13)	0.11001 (15)	0.0190 (3)	
C11	0.10054 (18)	0.05168 (13)	0.11525 (16)	0.0215 (4)	
H11	0.0391	0.0126	0.1559	0.026*	
C12	0.06711 (17)	0.13596 (14)	0.04673 (16)	0.0204 (4)	
C13	−0.05440 (18)	0.18738 (15)	0.01868 (16)	0.0256 (4)	
H13	−0.1397	0.1651	0.0442	0.031*	
C14	−0.0484 (2)	0.27035 (16)	−0.04612 (17)	0.0283 (4)	
H14	−0.1304	0.3053	−0.0655	0.034*	
C15	0.0767 (2)	0.30439 (15)	−0.08420 (16)	0.0281 (4)	
H15	0.0783	0.3624	−0.1277	0.034*	
C16	0.19806 (19)	0.25458 (14)	−0.05925 (16)	0.0238 (4)	
H16	0.2827	0.2777	−0.0852	0.029*	
C17	0.19268 (17)	0.16992 (13)	0.00483 (15)	0.0188 (3)	
C1A	0.14884 (18)	0.38445 (14)	0.24981 (15)	0.0219 (4)	
H1D	0.1228	0.3324	0.1924	0.026*	
H1E	0.1900	0.4361	0.2037	0.026*	
C2A	0.05447 (18)	0.51442 (13)	0.36912 (15)	0.0216 (4)	
H2D	0.1376	0.5072	0.4258	0.026*	
H2E	−0.0229	0.5309	0.4187	0.026*	
C3A	0.0777 (2)	0.59665 (15)	0.28370 (18)	0.0299 (4)	
H3D	0.0966	0.6543	0.3317	0.045*	
H3E	−0.0047	0.6061	0.2284	0.045*	
H3F	0.1563	0.5826	0.2359	0.045*	
C4A	−0.09529 (19)	0.42606 (15)	0.21401 (16)	0.0252 (4)	
H4D	−0.1106	0.3629	0.1764	0.030*	
H4E	−0.0714	0.4704	0.1490	0.030*	
C5A	−0.2272 (2)	0.45879 (17)	0.26472 (19)	0.0327 (5)	
H5D	−0.3030	0.4539	0.2013	0.049*	
H5E	−0.2173	0.5248	0.2914	0.049*	
H5F	−0.2470	0.4190	0.3343	0.049*	
C6A	−0.0962 (2)	0.21207 (14)	0.36621 (17)	0.0259 (4)	
H6D	−0.1936	0.2277	0.3485	0.039*	
H6E	−0.0880	0.1582	0.4225	0.039*	
H6F	−0.0553	0.1955	0.2902	0.039*	
C7A	−0.06954 (18)	0.37556 (15)	0.58896 (16)	0.0234 (4)	
H7D	−0.1698	0.3820	0.5775	0.035*	
H7E	−0.0281	0.4380	0.6048	0.035*	
H7F	−0.0456	0.3337	0.6585	0.035*	
C10A	0.25049 (18)	0.34955 (13)	0.34895 (15)	0.0198 (3)	
C11A	0.39089 (18)	0.34640 (14)	0.36008 (16)	0.0223 (4)	
H11A	0.4520	0.3707	0.3042	0.027*	
C12A	0.42739 (18)	0.29896 (12)	0.47316 (16)	0.0202 (4)	
C13A	0.55142 (18)	0.27146 (14)	0.53626 (18)	0.0261 (4)	
H13A	0.6369	0.2843	0.5032	0.031*	
C14A	0.54780 (19)	0.22547 (15)	0.64699 (18)	0.0284 (4)	
H14A	0.6316	0.2067	0.6899	0.034*	
C15A	0.4220 (2)	0.20598 (14)	0.69735 (17)	0.0256 (4)	
H15A	0.4225	0.1744	0.7737	0.031*	
C16A	0.29816 (18)	0.23198 (13)	0.63774 (16)	0.0205 (3)	
H16A	0.2135	0.2192	0.6723	0.025*	
C17A	0.30091 (17)	0.27766 (13)	0.52507 (15)	0.0179 (3)	

### Atomic displacement parameters (Å^2^)

**Table:**

	*U*^11^	*U*^22^	*U*^33^	*U*^12^	*U*^13^	*U*^23^
Al1	0.0165 (2)	0.0158 (3)	0.0194 (2)	0.00128 (19)	0.00356 (18)	0.00057 (19)
Al1A	0.0162 (2)	0.0184 (3)	0.0150 (2)	−0.00063 (19)	0.00145 (18)	−0.00094 (19)
N1	0.0183 (6)	0.0191 (8)	0.0189 (7)	0.0012 (6)	0.0020 (5)	0.0021 (6)
N1A	0.0193 (7)	0.0195 (8)	0.0168 (6)	0.0008 (6)	0.0031 (5)	0.0009 (6)
N2	0.0177 (6)	0.0129 (7)	0.0192 (6)	0.0013 (6)	0.0003 (5)	−0.0014 (6)
N2A	0.0219 (7)	0.0181 (7)	0.0152 (6)	0.0007 (6)	0.0000 (5)	−0.0016 (5)
C1	0.0208 (8)	0.0148 (8)	0.0225 (8)	−0.0028 (7)	0.0007 (6)	0.0005 (6)
C2	0.0212 (8)	0.0159 (9)	0.0258 (8)	0.0046 (7)	−0.0001 (7)	0.0011 (7)
C3	0.0231 (9)	0.0283 (11)	0.0409 (11)	0.0037 (8)	−0.0039 (8)	0.0035 (9)
C4	0.0265 (8)	0.0148 (9)	0.0191 (8)	0.0010 (7)	0.0022 (6)	−0.0021 (6)
C5	0.0516 (12)	0.0236 (10)	0.0216 (9)	−0.0026 (9)	0.0099 (8)	−0.0019 (8)
C6	0.0255 (9)	0.0194 (10)	0.0348 (10)	−0.0016 (7)	0.0083 (8)	0.0002 (8)
C7	0.0277 (9)	0.0250 (10)	0.0241 (9)	0.0055 (8)	0.0061 (7)	0.0005 (7)
C10	0.0209 (8)	0.0165 (8)	0.0194 (7)	−0.0024 (7)	0.0012 (6)	−0.0009 (6)
C11	0.0194 (8)	0.0227 (10)	0.0225 (8)	−0.0037 (7)	0.0024 (6)	−0.0004 (7)
C12	0.0194 (8)	0.0235 (9)	0.0182 (8)	0.0007 (7)	0.0011 (6)	−0.0046 (7)
C13	0.0211 (8)	0.0341 (11)	0.0216 (8)	0.0050 (8)	0.0013 (7)	−0.0061 (7)
C14	0.0283 (9)	0.0345 (11)	0.0216 (8)	0.0148 (8)	−0.0018 (7)	−0.0042 (8)
C15	0.0390 (10)	0.0253 (10)	0.0201 (8)	0.0117 (8)	0.0026 (7)	0.0031 (7)
C16	0.0275 (9)	0.0256 (10)	0.0189 (8)	0.0042 (7)	0.0048 (7)	0.0027 (7)
C17	0.0206 (8)	0.0206 (9)	0.0152 (7)	0.0024 (7)	0.0011 (6)	−0.0019 (6)
C1A	0.0253 (8)	0.0244 (9)	0.0166 (8)	0.0014 (7)	0.0053 (6)	0.0000 (7)
C2A	0.0268 (8)	0.0181 (9)	0.0197 (8)	−0.0009 (7)	0.0006 (7)	−0.0026 (7)
C3A	0.0372 (10)	0.0223 (10)	0.0301 (9)	−0.0035 (8)	0.0034 (8)	0.0017 (8)
C4A	0.0296 (9)	0.0271 (10)	0.0177 (8)	−0.0012 (8)	−0.0064 (7)	0.0010 (7)
C5A	0.0275 (9)	0.0354 (12)	0.0338 (10)	0.0040 (8)	−0.0064 (8)	0.0004 (9)
C6A	0.0291 (9)	0.0246 (10)	0.0237 (9)	−0.0051 (8)	−0.0004 (7)	−0.0022 (8)
C7A	0.0211 (8)	0.0295 (10)	0.0198 (8)	−0.0006 (7)	0.0034 (6)	−0.0037 (7)
C10A	0.0239 (8)	0.0183 (8)	0.0177 (8)	−0.0005 (7)	0.0063 (6)	−0.0015 (6)
C11A	0.0225 (8)	0.0211 (9)	0.0244 (8)	−0.0027 (7)	0.0097 (7)	−0.0041 (7)
C12A	0.0209 (8)	0.0158 (9)	0.0242 (8)	−0.0003 (6)	0.0050 (7)	−0.0067 (6)
C13A	0.0182 (8)	0.0244 (10)	0.0358 (10)	0.0015 (7)	0.0040 (7)	−0.0085 (8)
C14A	0.0227 (9)	0.0277 (10)	0.0335 (10)	0.0079 (7)	−0.0063 (7)	−0.0081 (8)
C15A	0.0307 (9)	0.0213 (10)	0.0239 (8)	0.0056 (8)	−0.0031 (7)	−0.0024 (7)
C16A	0.0226 (8)	0.0172 (9)	0.0218 (8)	0.0021 (7)	0.0028 (6)	−0.0024 (6)
C17A	0.0188 (8)	0.0148 (8)	0.0203 (8)	0.0008 (6)	0.0021 (6)	−0.0047 (7)

### Geometric parameters (Å, º)

**Table:**

Al1—N1	1.8879 (14)	C12—C17	1.422 (2)
Al1—C6	1.957 (2)	C13—C14	1.375 (3)
Al1—C7	1.9686 (19)	C13—H13	0.9500
Al1—N2	2.0355 (15)	C14—C15	1.403 (3)
Al1A—N1A	1.8779 (15)	C14—H14	0.9500
Al1A—C6A	1.960 (2)	C15—C16	1.386 (3)
Al1A—C7A	1.9610 (18)	C15—H15	0.9500
Al1A—N2A	2.0397 (16)	C16—C17	1.391 (3)
N1—C10	1.382 (2)	C16—H16	0.9500
N1—C17	1.384 (2)	C1A—C10A	1.501 (2)
N1A—C17A	1.378 (2)	C1A—H1D	0.9900
N1A—C10A	1.384 (2)	C1A—H1E	0.9900
N2—C2	1.499 (2)	C2A—C3A	1.521 (3)
N2—C1	1.504 (2)	C2A—H2D	0.9900
N2—C4	1.510 (2)	C2A—H2E	0.9900
N2A—C4A	1.501 (2)	C3A—H3D	0.9800
N2A—C2A	1.509 (2)	C3A—H3E	0.9800
N2A—C1A	1.509 (2)	C3A—H3F	0.9800
C1—C10	1.500 (2)	C4A—C5A	1.511 (3)
C1—H1A	0.9900	C4A—H4D	0.9900
C1—H1B	0.9900	C4A—H4E	0.9900
C2—C3	1.515 (3)	C5A—H5D	0.9800
C2—H2A	0.9900	C5A—H5E	0.9800
C2—H2B	0.9900	C5A—H5F	0.9800
C3—H3A	0.9800	C6A—H6D	0.9800
C3—H3B	0.9800	C6A—H6E	0.9800
C3—H3C	0.9800	C6A—H6F	0.9800
C4—C5	1.520 (2)	C7A—H7D	0.9800
C4—H4A	0.9900	C7A—H7E	0.9800
C4—H4B	0.9900	C7A—H7F	0.9800
C5—H5A	0.9800	C10A—C11A	1.366 (2)
C5—H5B	0.9800	C11A—C12A	1.433 (3)
C5—H5C	0.9800	C11A—H11A	0.9500
C6—H6A	0.9800	C12A—C13A	1.404 (2)
C6—H6B	0.9800	C12A—C17A	1.428 (2)
C6—H6C	0.9800	C13A—C14A	1.382 (3)
C7—H7A	0.9800	C13A—H13A	0.9500
C7—H7B	0.9800	C14A—C15A	1.409 (3)
C7—H7C	0.9800	C14A—H14A	0.9500
C10—C11	1.365 (2)	C15A—C16A	1.380 (2)
C11—C12	1.433 (3)	C15A—H15A	0.9500
C11—H11	0.9500	C16A—C17A	1.398 (2)
C12—C13	1.404 (2)	C16A—H16A	0.9500
			
N1—Al1—C6	111.91 (8)	C14—C13—C12	119.17 (18)
N1—Al1—C7	116.33 (8)	C14—C13—H13	120.4
C6—Al1—C7	117.73 (8)	C12—C13—H13	120.4
N1—Al1—N2	85.25 (6)	C13—C14—C15	121.16 (17)
C6—Al1—N2	115.96 (7)	C13—C14—H14	119.4
C7—Al1—N2	105.14 (7)	C15—C14—H14	119.4
N1A—Al1A—C6A	113.03 (8)	C16—C15—C14	120.99 (19)
N1A—Al1A—C7A	114.12 (7)	C16—C15—H15	119.5
C6A—Al1A—C7A	118.00 (8)	C14—C15—H15	119.5
N1A—Al1A—N2A	85.91 (6)	C15—C16—C17	118.25 (17)
C6A—Al1A—N2A	108.30 (7)	C15—C16—H16	120.9
C7A—Al1A—N2A	112.91 (8)	C17—C16—H16	120.9
C10—N1—C17	105.83 (13)	N1—C17—C16	129.35 (16)
C10—N1—Al1	112.84 (11)	N1—C17—C12	109.32 (16)
C17—N1—Al1	139.57 (13)	C16—C17—C12	121.28 (16)
C17A—N1A—C10A	106.15 (14)	C10A—C1A—N2A	108.11 (13)
C17A—N1A—Al1A	140.50 (12)	C10A—C1A—H1D	110.1
C10A—N1A—Al1A	113.18 (11)	N2A—C1A—H1D	110.1
C2—N2—C1	108.22 (13)	C10A—C1A—H1E	110.1
C2—N2—C4	112.02 (12)	N2A—C1A—H1E	110.1
C1—N2—C4	110.43 (13)	H1D—C1A—H1E	108.4
C2—N2—Al1	115.63 (10)	N2A—C2A—C3A	115.85 (14)
C1—N2—Al1	101.69 (10)	N2A—C2A—H2D	108.3
C4—N2—Al1	108.35 (10)	C3A—C2A—H2D	108.3
C4A—N2A—C2A	112.02 (14)	N2A—C2A—H2E	108.3
C4A—N2A—C1A	109.27 (13)	C3A—C2A—H2E	108.3
C2A—N2A—C1A	110.02 (13)	H2D—C2A—H2E	107.4
C4A—N2A—Al1A	114.30 (11)	C2A—C3A—H3D	109.5
C2A—N2A—Al1A	108.47 (10)	C2A—C3A—H3E	109.5
C1A—N2A—Al1A	102.31 (11)	H3D—C3A—H3E	109.5
C10—C1—N2	107.43 (14)	C2A—C3A—H3F	109.5
C10—C1—H1A	110.2	H3D—C3A—H3F	109.5
N2—C1—H1A	110.2	H3E—C3A—H3F	109.5
C10—C1—H1B	110.2	N2A—C4A—C5A	113.37 (15)
N2—C1—H1B	110.2	N2A—C4A—H4D	108.9
H1A—C1—H1B	108.5	C5A—C4A—H4D	108.9
N2—C2—C3	113.28 (15)	N2A—C4A—H4E	108.9
N2—C2—H2A	108.9	C5A—C4A—H4E	108.9
C3—C2—H2A	108.9	H4D—C4A—H4E	107.7
N2—C2—H2B	108.9	C4A—C5A—H5D	109.5
C3—C2—H2B	108.9	C4A—C5A—H5E	109.5
H2A—C2—H2B	107.7	H5D—C5A—H5E	109.5
C2—C3—H3A	109.5	C4A—C5A—H5F	109.5
C2—C3—H3B	109.5	H5D—C5A—H5F	109.5
H3A—C3—H3B	109.5	H5E—C5A—H5F	109.5
C2—C3—H3C	109.5	Al1A—C6A—H6D	109.5
H3A—C3—H3C	109.5	Al1A—C6A—H6E	109.5
H3B—C3—H3C	109.5	H6D—C6A—H6E	109.5
N2—C4—C5	115.68 (15)	Al1A—C6A—H6F	109.5
N2—C4—H4A	108.4	H6D—C6A—H6F	109.5
C5—C4—H4A	108.4	H6E—C6A—H6F	109.5
N2—C4—H4B	108.4	Al1A—C7A—H7D	109.5
C5—C4—H4B	108.4	Al1A—C7A—H7E	109.5
H4A—C4—H4B	107.4	H7D—C7A—H7E	109.5
C4—C5—H5A	109.5	Al1A—C7A—H7F	109.5
C4—C5—H5B	109.5	H7D—C7A—H7F	109.5
H5A—C5—H5B	109.5	H7E—C7A—H7F	109.5
C4—C5—H5C	109.5	C11A—C10A—N1A	112.32 (15)
H5A—C5—H5C	109.5	C11A—C10A—C1A	132.80 (16)
H5B—C5—H5C	109.5	N1A—C10A—C1A	114.84 (14)
Al1—C6—H6A	109.5	C10A—C11A—C12A	106.03 (15)
Al1—C6—H6B	109.5	C10A—C11A—H11A	127.0
H6A—C6—H6B	109.5	C12A—C11A—H11A	127.0
Al1—C6—H6C	109.5	C13A—C12A—C17A	118.82 (17)
H6A—C6—H6C	109.5	C13A—C12A—C11A	135.04 (17)
H6B—C6—H6C	109.5	C17A—C12A—C11A	106.14 (15)
Al1—C7—H7A	109.5	C14A—C13A—C12A	119.22 (17)
Al1—C7—H7B	109.5	C14A—C13A—H13A	120.4
H7A—C7—H7B	109.5	C12A—C13A—H13A	120.4
Al1—C7—H7C	109.5	C13A—C14A—C15A	121.12 (17)
H7A—C7—H7C	109.5	C13A—C14A—H14A	119.4
H7B—C7—H7C	109.5	C15A—C14A—H14A	119.4
C11—C10—N1	112.64 (15)	C16A—C15A—C14A	121.18 (18)
C11—C10—C1	132.87 (16)	C16A—C15A—H15A	119.4
N1—C10—C1	114.36 (14)	C14A—C15A—H15A	119.4
C10—C11—C12	105.78 (15)	C15A—C16A—C17A	118.04 (17)
C10—C11—H11	127.1	C15A—C16A—H16A	121.0
C12—C11—H11	127.1	C17A—C16A—H16A	121.0
C13—C12—C17	119.11 (18)	N1A—C17A—C16A	129.05 (16)
C13—C12—C11	134.47 (17)	N1A—C17A—C12A	109.34 (15)
C17—C12—C11	106.40 (15)	C16A—C17A—C12A	121.61 (16)

### Hydrogen-bond geometry (Å, º)

Cg1 is the centroid of the C12*A*–C17*A* ring.

**Table:**

*D*—H···*A*	*D*—H	H···*A*	*D*···*A*	*D*—H···*A*
C5—H5*A*···*Cg*1	0.98	2.57	3.470 (2)	153
C2—H2*B*···*Cg*1^i^	0.99	2.55	3.434 (2)	149

Symmetry code: (i) −*x*+1, *y*−1/2, −*z*+1.
